# Multimodal Investigation of Angiogenesis and Its Prevention by Small Compounds in a Zebrafish Cancer Model

**DOI:** 10.1002/advs.202415176

**Published:** 2025-06-25

**Authors:** Marco Andreana, Ryan Sentosa, Caterina Sturtzel, Martin Pfister, René Werkmeister, Anna Schmitt, David Traver, Rainer Leitgeb, Wolfgang Drexler, Martin Distel, Angelika Unterhuber

**Affiliations:** ^1^ Center for Medical Physics and Biomedical Engineering Medical University of Vienna Vienna 1090 Austria; ^2^ Innovative Cancer Models St. Anna Children's Cancer Research Institute Vienna 1090 Austria; ^3^ Zebrafish Platform Austria for preclinical drug screening (ZANDR) Vienna 1090 Austria; ^4^ Laboratory of Experimental Immunology, Institute of Virology, Faculty of Medicine and University Hospital Cologne University Cologne 50935 Cologne Germany; ^5^ Department of Cell and Developmental Biology University of California San Diego La Jolla 92093 USA; ^6^ Division of Pediatric Hematology and Oncology, Intermountain Primary Children's Hospital, Huntsman Cancer Institute Spencer Fox Eccles School of Medicine at the University of Utah Salt Lake City 84113 USA

**Keywords:** angiographic imaging, cancer model, in vivo, label‐free, optical coherence tomography angiography, optical coherence tomography, topological descriptors

## Abstract

Aberrant angiogenesis is a hallmark of many pathologies. In cancer, tumor growth and metastasis strongly depend on angiogenesis triggered by neoplastic cells. Antiangiogenic therapies are approved to treat different kinds of cancer. However, the success of these treatments is so far limited as some patients do not respond at all and others develop resistances. Thus, a deeper understanding of the mechanisms driving tumor angiogenesis and the variations in tumor vessels is crucial. Optical coherence tomography angiography (OCTA) is a fast volumetric imaging technique that provides detailed insights into tumor vascularization and perfusion of vessels in a label‐free and non‐invasive manner. An ultra‐high resolution OCTA and confocal fluorescence imaging pipeline are developed to analyze tumor vascularization and blood perfusion in vivo, using a zebrafish cancer model. OCTA imaging operating at 800 nm is optimized to show slow blood flow allowing to compare the functionality of blood vessels in healthy and tumor‐bearing zebrafish. Furthermore, effects of small compounds on tumor vascularization can be investigated with our setup. The key outcomes include a qualitative assessment of vascularization and blood vessel perfusion, along with a quantitative analysis of vessel structure, to evaluate how effective the drugs were at different concentrations.

## Graphical abstract

A combined optical coherence tomography angiography and fluorescence imaging approach assesses tumor angiogenesis and drug effects in a novel zebrafish cancer model. By analyzing 3D vasculature data, the two imaging methods complement each other to quantify blood flow, vessel structure, and drug efficacy. Results highlight impaired perfusion in cancerous models and demonstrate potential for preclinical drug screening and vascular research.

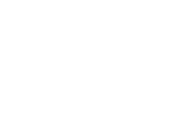

## 1 Introduction

Over the last three decades optical coherence tomography (OCT) has become the prime diagnostic imaging modality in ophthalmology^[^ ^1^, ^2^ ^]^ and has increased its impact into many other biomedical research fields including cancer research.^[^ ^3^, ^4^, ^5^ ^]^ OCT provides fast label‐free, non‐invasive, and high‐resolution volumetric structural information of tissue based on low‐coherence light interferometry. It enables spatial resolution down to microns over areas of a couple of cm^2^ with penetration depths up to several millimeters.^[^ ^6^, ^7^ ^]^ OCTA has emerged from OCT as functional extension and generates exquisitely detailed 3D angiograms at video rates. OCTA analyses the intrinsic dynamic changes in the OCT signal caused by moving particles such as red blood cells. The signal is based on the decorrelation of the cross‐sectional OCT signal (the B‐scans) repeatedly imaged at the same tissue location over time. As a result, static particles have high OCT signal correlation (low image pixel value) and moving particles have low OCT signal correlation (high image pixel value). By changing the time span between consecutive OCT B‐scans blood flow velocity maps are created, i.e., fast, slow, and no flow.^[^ ^8^, ^9^ ^]^

As fast and safe non‐contact and non‐ionizing method it has been increasingly applied in ocular diseases to assess the chorioretinal microvasculature and is rapidly expanding in clinical practice ocular oncology while its translation to the nonocular clinical oncology still poses certain limitations such as a lack of standardized protocols and interpretation guidelines.^[^ ^10^ ^]^ Blood vessels are the largest network in our body and the formation of new blood vessels contributes to numerous malignant, ischemic, inflammatory, infectious and immune disorders including diabetic retinopathy, cardiovascular disease, atherosclerosis, ischemia and delayed wound healing^[^ ^11^, ^12^ ^]^ when dysregulated. Deeper insights into these processes are required to offer new therapeutic opportunities for the long list of disorders that is characterized or caused by excessive angiogenesis.^[^ ^13^ ^]^ Angiogenesis is a complex chemically regulated process through which new vessels from pre‐existing functional ones are created. This multistep process involves migration, growth and differentiation of endothelial cells in order to form new vessels under specific chemical stimulations.^[^ ^14^, ^15^ ^]^ Angiogenesis occurs throughout the entire life in both healthy and pathologic conditions including tissue repair, organ regeneration and tumorigenesis and is responsible for the continuous remodeling of the vascular network. Triggered by certain growth factors, for instance with the vascular endothelial growth factor (VEGF) as one of the most specific and critical regulators of angiogenesis, different types of angiogenesis occur: sprouting and splitting or intussusceptive.^[^ ^16^, ^17^ ^]^ In sprouting angiogenesis endothelial cells grow toward a VEGF stimulus from existing vessels. Therefore, sprouting angiogenesis adds new blood vessels to the tissue where no blood vessels were previously present. In splitting angiogenesis, all endothelial cells respond to VEGF the same way thus resulting in circumferential vascular enlargement followed by the splitting of the original vessel into two.^[^ ^18^ ^]^

The chemical stimulation promoting tissue vascularization can be abducted by abnormal cells and presence of high VEGF concentrations is associated with specific pathological conditions. For various types of cancer pathological angiogenesis has become a hallmark and multiple mechanisms of tumor angiogenic processes have been identified.^[^ ^19^ ^]^ Tumors, composed of populations of abnormal cells with the ability to proliferate without any apparent control, rely even more than normal cells on high supply of oxygen and nutrients for all the aberrant metabolic processes. Therefore, tumor cells need to gain the capacity to induce their own blood supply by hijacking pathways of physiological angiogenesis.^[^ ^20^, ^21^ ^]^ This effect is called angiogenic switch. Access to blood vessels also provides an escape route to tumor cells and contributes to metastasis.^[^ ^22^ ^]^ Hence, the inhibition of angiogenic factors represents one of the strategies for cancer treatment but also for the prevention of cancer recurrence and metastasis.^[^ ^23^ ^]^ In recent years, the concept of the tumor microenvironment was born suggesting to view tumors as complex tissue, where cancer cells have co‐opted normal cells to collaborate in their neoplastic agenda. The tumor cells initiate important molecular, cellular and physical changes within their host tissue to assist tumor growth and progression. Hallmark components of the tumor microenvironment include immune cells, stroma cells, extracellular matrix and blood vessels. While angiogenic mechanisms are co‐opted during tumor vascularization, tumor blood vessels differ from normal vessels being irregularly shaped and often leaky.^[^ ^22^ ^]^ Importantly, only functional blood vessels can deliver therapeutics to the tumor. The important role of the tumor microvasculature in supporting tumor growth but also drug delivery and the complexity of the tumor microenvironment underscore the need to study the interactions of preneoplastic and neoplastic cells with immune cells and endothelial cells within their natural environment.^[^ ^24^ ^]^

Since angiogenesis is largely conserved among different vertebrate species, in vivo animal models have been established to provide a physiological model of angiogenesis and important mechanistic insights to tumor angiogenesis.^[^ ^25^ ^]^ Over the last decade, zebrafish (*Danio rerio*), a fresh water fish native to southeast Asia, has gained particular attention in preclinical research due to many advantages compared to classical in vitro assays and other vertebrate animal models.^[^ ^26^, ^27^ ^]^ High fecundity, easy breeding and modest costs make zebrafish an attractive model. In addition, zebrafish are fast growing and transparent, thus providing a unique opportunity to study this animal model with optical microscopy. Most importantly, zebrafish share up to 71 % of human genes and around 84 % of genes known to be associated with human diseases have a zebrafish counterpart.^[^ ^28^, ^29^ ^]^ Indeed, several zebrafish cancer models have been established, including leukemia, melanoma, neuroblastoma, liver, pancreatic, and testicular cancer.^[^ ^30^ ^]^ Due to the optical clarity of zebrafish larvae, confocal fluorescence laser scanning microscopy (cLSM) is the gold standard imaging method to observe transformed cell behavior and also cell interactions and processes like neo‐angiogenesis within the tumor microenvironment in the living organisms. However, an intrinsic limitation of confocal fluorescent imaging is the inability to reveal structures and flow from non‐fluorescent labeled tissue and cells.^[^ ^31^ ^]^ With the use of transgenic fluorescent zebrafish reporter lines monitoring angiogenesis in real‐time under cLSM longitudinal studies and follow ups over several days or large‐scale drug‐screenings are possible.^[^ ^26^, ^32^, ^33^ ^]^ Several transgenic fish strains, expressing different fluorescent proteins in endothelial cells or in erythrocytes are available for tracking angiogenesis.^[^ ^34^, ^35^ ^]^ In addition, fluorescent microspheres can be used to highlight the blood flow.^[^ ^36^ ^]^ Hence, the zebrafish as animal model provides important insights of the vasculature based on contrast agents or on transgenic models under non‐pathological and pathological conditions.^[^ ^32^, ^34^, ^37^ ^]^ Endothelial cells expressing fluorescent proteins can be tracked in live cultures. However, the expression of fluorescent proteins requires sophisticated genetic manipulation. On the other hand, staining using dyes is limited to the number of cell or organelle‐specific dyes and labeling is not permanent, but typically fades over several cell divisions.^[^ ^33^ ^]^ Furthermore, spectral overlap of both, dyes or fluorescent proteins is a common problem, preventing simultaneous observation of more than 3‐4 features of interest.

OCT^[^ ^6^, ^7^ ^]^ as label‐free technique does not rely on the use of exogenous agents. As consequence, OCT does not suffer from photobleaching and phototoxicity with release of singlet oxygen and free radicals as in cLSM. To avoid the use of extensive labeling or the development of multicolor transgenic lines, OCTA has been introduced as fast 3D flow imaging technique.^[^ ^4^, ^38^, ^39^, ^40^ ^]^

In this paper, we present a combined OCTA and cLSM approach to quantitatively assess tumor angiogenesis and the effect of different drug treatments in a zebrafish cancer model in vivo. In particular, we analyze 3D OCTA and cLSM data sets of the vasculature in our transgenic cancer model. The aim of this work is threefold: (1) produce 3D projections of zebrafish vasculature and compare OCTA contrast from moving blood cells with GFP expression in endothelial cells; (2) study angiogenesis in a cancer model and (3) elucidate the effects of different drug treatments on the progression of uncontrolled microangiogenesis. In detail, we topologically and quantitatively analyze the microvasculature in OCTA and GFP angiograms of zebrafish larvae to objectively evaluate differences between a control group (healthy), cancerous and larvae treated with different targeted inhibitors. We also perform dose dependent compound testing and evaluated effects.

## 2 Results

### 2.1 A Larval Zebrafish Cancer Model With Aberrant Vasculature

**Figure 1:**
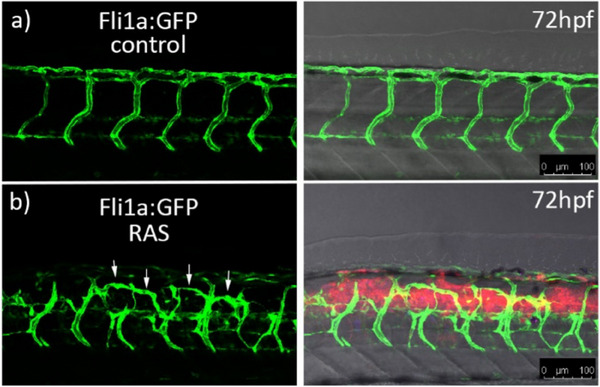
Vascular abnormalities in RAS‐expressing zebrafish: a) Trunk region of a transgenic zebrafish (*Tg(fli1a:EGFP)* ^*y*1^) expressing EGFP in endothelial cells at 72 hpf. Evenly spaced intersegmental vessels are visible. Overlay of brightfield and fluorescence (right); b) Trunk region of a transgenic zebrafish expressing HRAS^*G*12*V*^ in the spinal cord (red) at 72hpf. Overlay of brightfield and two fluorescent channels (right). Hyperbranching (arrows) of vessels (green) between intersegmental vessels is visible. Images on the right are overlays between fluorescence and brightfield images (grey). Scale bar is 100 µM.

We established a larval zebrafish cancer model with altered vasculature surrounding oncogene expressing regions. For this, we used the Gal4/UAS system to target oncogenic HRAS^*G*12*V*^ to the developing central nervous system (CNS).^[^ ^41^ ^]^ This was achieved by crossing the SP8b:KalTA4 activator strain, which shows expression of a zebrafish optimized Gal4 variant (KalTA4) throughout the CNS including the spinal cord and *Tg*(*H2BCFP:UAS: HRAS* ^*G*12*V*^), an effector strain for co‐expression of the blue nuclei marker H2B‐CFP and human constitutively active HRAS^*G*12*V*^. This results in HRAS^*G*12*V*^ expressing cells hyper‐proliferating in the spinal cord. To visualize potential effects of tumor cells on the vasculature, we generated triple transgenic zebrafish *Tg(fli1a:EGFP)/Et(SP8b:KalTA4‐1xUAS:mCherry)/Tg*(*H2BCFP:UAS: HRAS* ^*G*12*V*^), where the vasculature is highlighted by GFP expression. This revealed that the vasculature surrounding oncogene‐expressing cells is dramatically altered at 72 h post fertilization (hpf) (**Figure** **1**).

**Figure 2:**
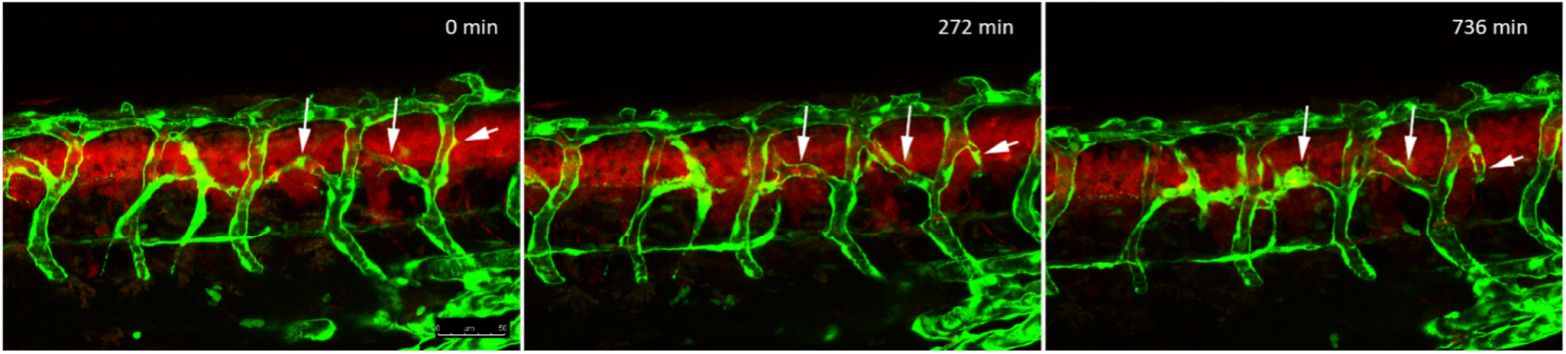
Hyperbranching in RAS‐expressing zebrafish. Stand stills from a time‐lapse movie starting around 57 hpf. Vascular sprouts develop from existing intersegmental vessels (arrows) and form new connections. Vessels shown in green and cells of the spinal cord expressing HRAS^*G*12*V*^ shown in red.

Time‐lapse imaging showed that intersegmental vessels initially develop normally, however at later stages cells start to sprout from existing vessels and establish new branches, which connect adjacent intersegmental vessels (**Figure** **2** (white arrows) and **Movie 1**). Although we could clearly visualize the formation of hyperbranches, it was unclear, if some or all of these new vessels are functional and show blood flow. Here, as we already used three fluorescent proteins to demarcate tumor cells (CFP and mCherry) and vessels (GFP), ideally a compatible method for label‐free visualization of blood flow is needed. Toward this goal, we developed an OCTA/cLSM platform. From now on we will refer to oncogene‐expressing and healthy larvae (control) as “RAS+” and “RAS‐” to indicate the presence or absence of constitutively active RAS.

### 2.2 Multimodal Optical Imaging Platform and Pipeline

**Figure 3:**
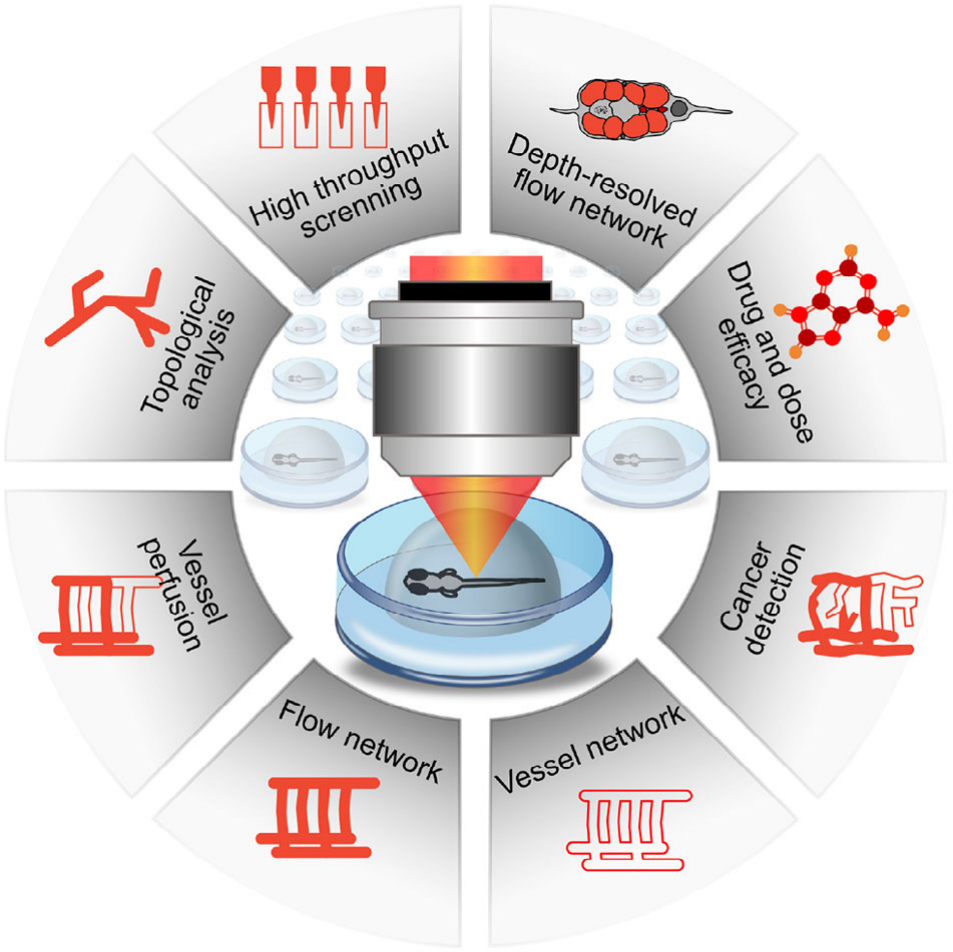
Overview of features that can be obtained in the context of vascular zebrafish imaging. The combination of OCT and fluorescence imaging allow high throughput screening for topological and vessel perfusion analysis, flow and vessel network visualization. Cancer development can be observed and compounds efficiency studies can be performed. By means of OCTA depth‐resolved flow network images are generated.

Initially, we designed the multimodal optical imaging setup to overcome the challenges associated with the evaluation of vasculature and vessel perfusion in the zebrafish. The multimodal imaging pipeline including OCTA and cLSM was optimized for imaging vasculature in zebrafish expressing GFP in the endothelial cells of (*Tg(fli1a:EGFP)* ^*y*1^) fish up to 120 hpf. **Figure** **3** highlights the features that can be extracted with our combination of OCTA and cLSM for high throughput zebrafish larvae screening of the vasculature network. OCTA offers fast 3D depth‐resolved information about the flow network that can be topologically analyzed. cLSM as gold standard technique for zebrafish imaging reveals additional complementary information about the arrangement of the vessel network. The combination of OCTA and GFP fluorescence data sets shows vessel perfusion and offers the unique opportunity to non‐destructively, non‐invasively study and analyze angiography maps. Since blood vessels are an important hallmark of the tumor microenvironment and the regulation of angiogenesis is disrupted during tumor formation, we investigated the neo‐angiogenesis process in our zebrafish cancer model with our imaging pipeline. In fact, our combination of OCTA and fluorescence imaging facilitates the study of cancer‐associated changes of angiogenesis and the effect of therapeutic intervention in a dose‐dependent manner. We used transgenic zebrafish strains based on RAS oncogene expression throughout the CNS including the spinal cord as detailed in Section 4. OCT, with its functional extension OCTA, offers the motion contrast, e.g., arising from the red blood cells while cLSM offers contrast from endothelial cells expressing enhanced GFP in the transgenic zebrafish. We optimized our OCTA platform in terms of acquisition speed, resolution, depth penetration and post processing for our zebrafish cancer model to optimally and non‐invasively highlight relevant biomarkers, such as blood flow and vessel networks as described in Section 2.3. We studied angiogenesis, pathological vascular alterations, and drug and dose dependent changes in 120 hpf larvae since profound changes of the cardiovascular system occur during this early developmental period.

**Figure 4:**
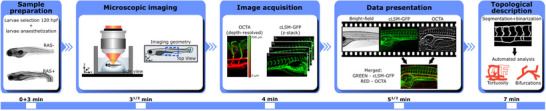
OCTA sequential imaging workflow. Sample preparation and selection are performed as first step in order to anaesthetize the larvae. Then, phytagel is used to fix the selected larvae in space for imaging over a glass dish filled with E3 medium. Next OCT depth resolved images and fluorescence GFP microscopy maps are recorded and OCTA images are calculated. Before any topological analysis can take place, images need to be co‐registered and a merged color‐coded image is created. The total imaging and analysis session take about 7 min per larvae.

In detail, the multimodal imaging workflow is shown in **Figure** **4**. Zebrafish larvae were manually selected and anaesthetized. 0.7% Phytagel was used to prevent movement of the selected larvae and imaging was performed in a glass dish filled with E3 medium. The zebrafish larvae were positioned with an automatized three axis stage and raster scanned with a galvanometric mirror pair such that an A‐scan covered the coronal plane of the zebrafish and the B‐scan the sagittal plane of the larvae from the anterior to the caudal end. Volumetric data sets were recorded and processed with a total imaging and analysis time of about seven minutes starting from the selection of the larvae. The volumetric OCT data produced label‐free angiography maps of the full zebrafish larvae and confocal fluorescence z‐scans were additionally acquired in order to visualize volumetric GFP distribution. Bright field images of the larvae revealed the phenotype. OCTA angiograms were obtained from maximum intensity projections across the full zebrafish thickness. These maximum projection en face images were merged and co‐registered with z‐stacked images from the GFP fluorescence signal to combine and evaluate the complementary information carried by the two imaging modalities. Merged color‐coded red green images were produced in order to highlight differences and similarities between flow and vasculature network. Topological analysis on defined regions‐of‐interest (ROI) was performed to objectively reveal associations between OCTA angiograms and pathology as well as drug response. Further details can be found in Section 4.

### 2.3 OCTA Imaging Optimization

**Figure 5:**
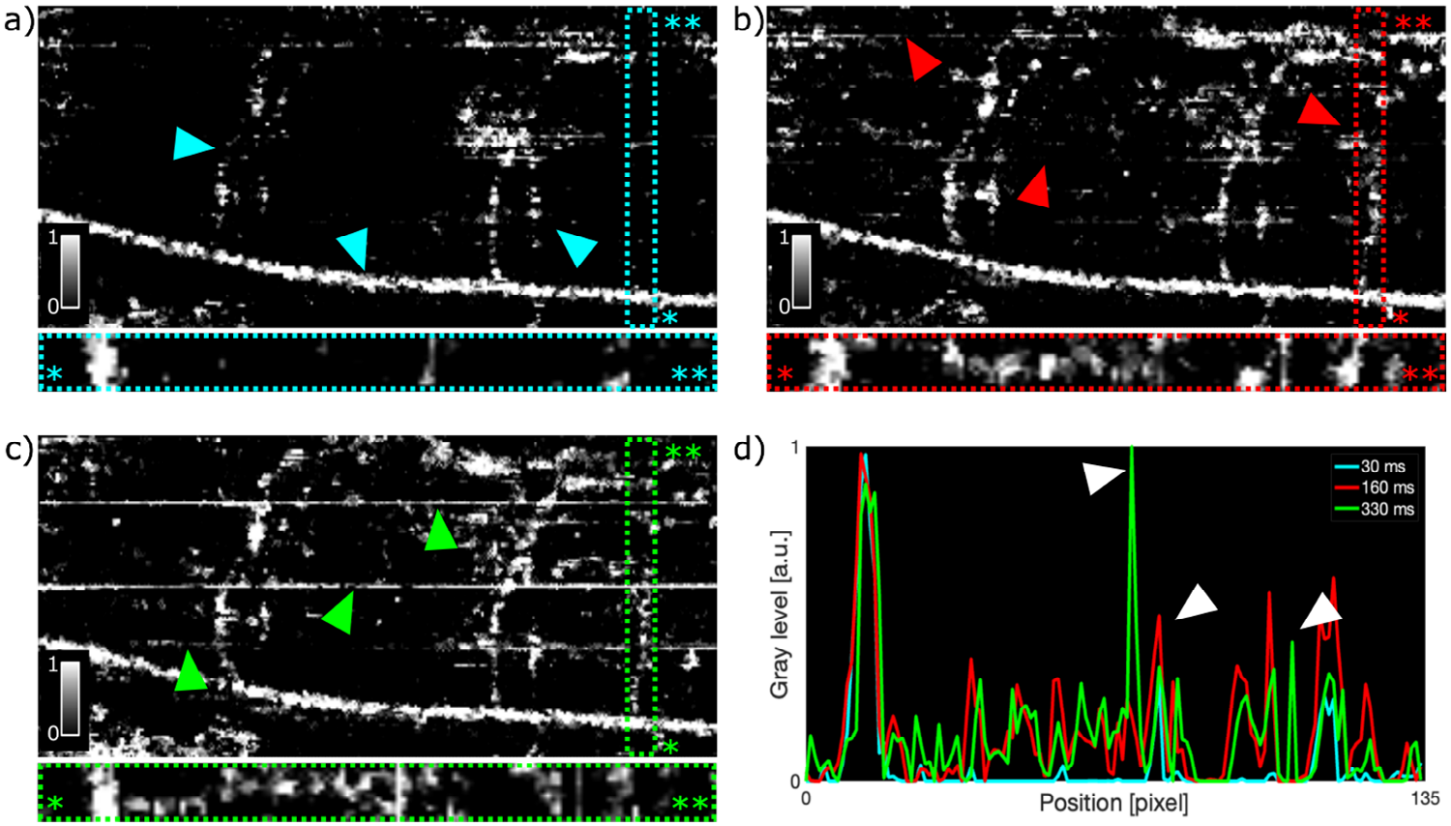
In vivo angiograms of the trunk of RAS+ zebrafish larvae at 120 hpf for different inter B‐scan times. a) B‐scan time of 30 ms. Cyan triangles indicate the position of DA and intersegmental vessels. b) 167 ms. Red triangles indicate the position of weak motion artifacts. c) 333 ms. Green triangles indicate the position of strong motion artifacts. d) Intensity profile of the slow flowing vessel in the framed area of a–c). White triangles highlight the motion artifacts for inter B‐scan time of 333 ms and 167 ms. The OCTA images in a–c) did not undergo data filtering. FOV is 487x190 µm^2^.

We have investigated the optimal inter B‐scan required to highlight large flow impairments present especially in cancerous systems such as RAS+ zebrafish larvae. Results are shown in **Figure** **5** for a 120 hpf RAS+ larvae. We have recorded 50 B‐scans per transversal region over the area corresponding to the fish trunk, with an A‐scan rate of 66.6 kHz and a minimum time interval of ∼30 ms. From the OCTA acquisition, we generated OCTA images using variable inter B‐scan time of ∼30, ∼160, and ∼330 ms. The OCTA data generated with ∼30 ms inter B‐scan rate (Figure 5a) are mostly sensitive to fast flow, visualizing the dorsal aorta (DA) with high contrast, and barely to the slow flows appearing in the intersegmental vessels (cyan triangles). When increasing the inter B‐scan rate to ∼160 ms (Figure 5b), OCTA shows contrast from dorsal aorta and an increased sensitivity for the slow flowing in the intersegmental vessels. The intersegmental vessels are much more visible in respect to the OCTA data using ∼ 30 ms interscan time. This increased inter B‐scan time allows the visualization of even slower flowing vessel as highlighted by the red dashed rectangle. Unwanted motion artifacts and background contrast are more visible with longer interscan time (red triangles). When increasing the interscan time even more to ∼330 ms, motion artifacts and background noise overwhelm the flow contrast (green triangles). The profile of the slow flowing vessel in the framed area is plotted for comparison between the three‐time intervals as shown in Figure 5d. The profile for the ∼30 ms time interval shows high contrast for the DA and almost no other contrasts. Changing the interscan time to ∼160 ms and ∼330 ms significantly improves the visualization of the slow flows but still keeps similar contrast for the DA. Motion artifacts and Brownian motion become more prominent with longer interscan times and result in local enhanced contrast contributing to an increased background contrast as highlighted in Figure 5d with white triangles. We identified ∼160 ms as the best compromise between good visualization of the slow flows and reduced presence of artifacts in the OCTA images and performed the measurement using this optimized parameter.

### 2.4 In Vivo Vascular Imaging

**Figure 6:**
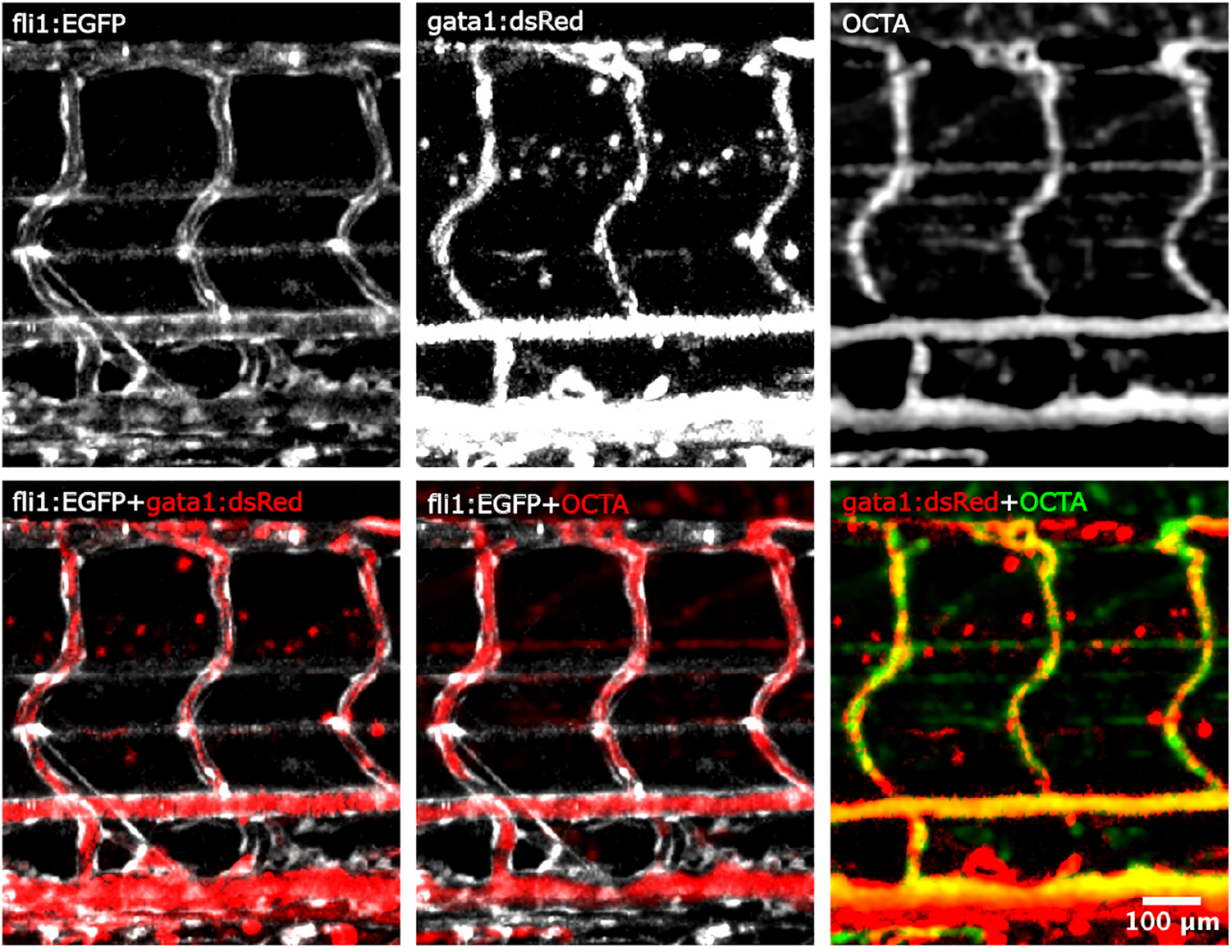
Vasculature images of fli1:EGFP reporter, gata1:dsRed erythrocytes reporter and OCTA. The overlay of the images shows the origin of the three signals for a 120 hpf larva.

We validated the origin of the OCTA signal by directly comparing it with a zebrafish transgenic line that expresses a fluorescent marker in red blood cells (gata1:dsRed). The results shown in **Figure** **6** are obtained from fluorescence and OCTA imaging of *fli1:EGFP/gata1:dsRed* double‐transgenic zebrafish. These images reveal the movement of erythrocytes within blood vessels, visualized through dsRed and GFP signals, and show strong overlap between the OCTA and dsRed signals in the intersegmental vessels and the dorsal aorta. In the venous plexus, the OCTA signal closely correlates with the dsRed signal from erythrocytes in the dorsal region. However, the reduced OCTA signal in the ventral part of the venous plexus suggests that the *gata1:dsRed*‐positive cells in this area are not moving. Additionally, we observed an OCTA signal within the neural tube, which we believe likely represents cilia‐mediated cerebrospinal fluid movement in the neural canal.

**Figure 7:**
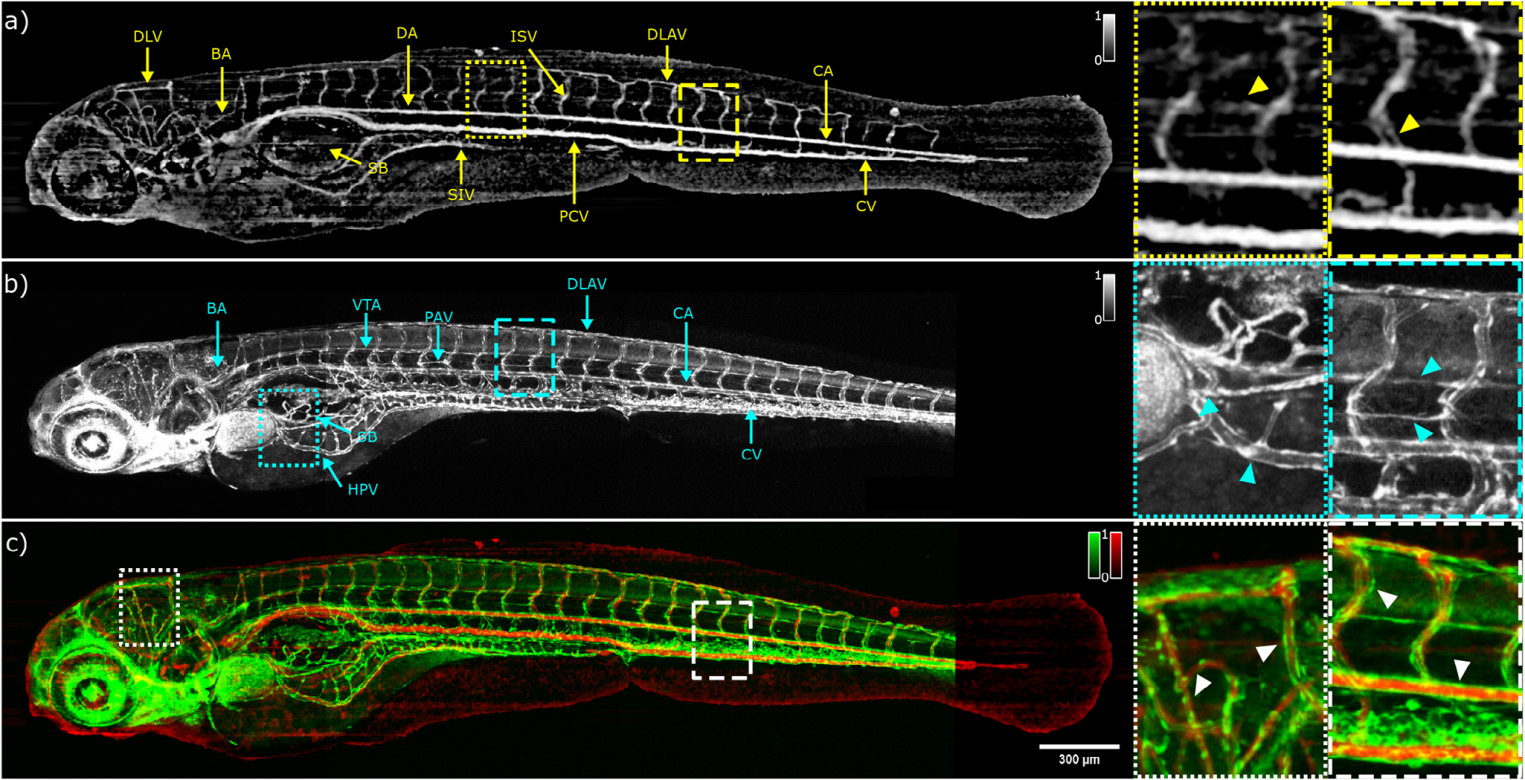
In vivo angiograms of RAS‐ zebrafish larvae at 120 hpf. a) en face label‐free OCTA image. The yellow triangle in the yellow dotted rectangle indicates the vertebral artery. The yellow triangle in the yellow dashed rectangle highlights the front and back intersegmental vessels. b) en face confocal GFP fluorescence image. The cyan triangles in the cyan dotted rectangle indicates the hepatic portal vein and pectoral artery location. The cyan triangles in the cyan dashed rectangle indicates the location of the parachordal vessel and the vertebral artery. c) merged image of the images in panel a) and b): red OCTA; green GFP fluorescence. The white triangles in the dotted and dashed rectangles show the complementary information of OCTA and cLSM. DLV, dorsal longitudinal vein; BA, basilar artery; DA, dorsal aorta; SIV, subintestinal vein; ISV, intersegmental vessels; PCV, posterior cardinal vein; DLAV, dorsal longitudinal anastomotic vessel; CA, caudal artery; CV, caudal vein; HPV, hepatic portal vein; SB, swim bladder; VTA, vertebral artery; PAV, parachordal vessel.

Representative label‐free OCTA and GFP labelled cLSM angiograms from a control zebrafish larva (RAS‐) are shown in **Figure** **7**. The well‐organized vascular network is already established at 120 hpf. It is clearly visible in the en face phase contrast image and all main vessels in head, trunk and tail are clearly distinguishable. The total body length is around 3.9 mm. Figure 7a shows the en face visualization of the maximum intensity OCTA projection. Following the vascular anatomy of the developing zebrafish as in Ref. [42], the main axial vessels covering the trunk and tail regions of zebrafish larvae at 120 hpf can be identified in the OCT angiogram: DA, caudal artery (CA), posterior cardinal vein (PCV) and caudal vein (CV). Intersegmental vessels (ISVs) elongating dorsally up from the DA and PCV are distinguishable. The sprout forms a “T” shape in the most dorsal region of the trunk to form a right and left pair of dorsal longitudinal anastomotic vessels (DLAVs). From this pair front and back ISVs are formed as shown in the inset (yellow dashed ROI) of Figure 7a. In the head region, main vessels are visible. Among all the complicated brain vessels' network the dorsal longitudinal vein (DLV) and the basilary artery (BA) can be clearly identified. The BA elongates longitudinally in order to supply blood to the spinal cord and forms a longitudinal vessel beneath the spinal cord and adjacent to the myotome called vertebral artery which is highlighted by OCTA as shown in the inset of Figure 7a (yellow dotted rectangle). Data from cLSM shown in Figure 7b highlight the vessel wall network of the zebrafish larvae and confirm the origin of the OCTA signals. The main axial vessels in the head and trunk are visible in this representation. The ISVs are connected to the CV and CA and show a regular pattern. Moreover, veins and arteries interconnecting the digestive tract are well distinguishable and the hepatic portal vein (HPV) can be easily identified. The pectoral artery (PA) and the HPV are highlighted in the cyan dotted ROI in Figure 7b. Furthermore, the parachordal vessel and the vertebral artery are shown in the cyan dashed ROI in Figure 7b.

**Figure 8:**
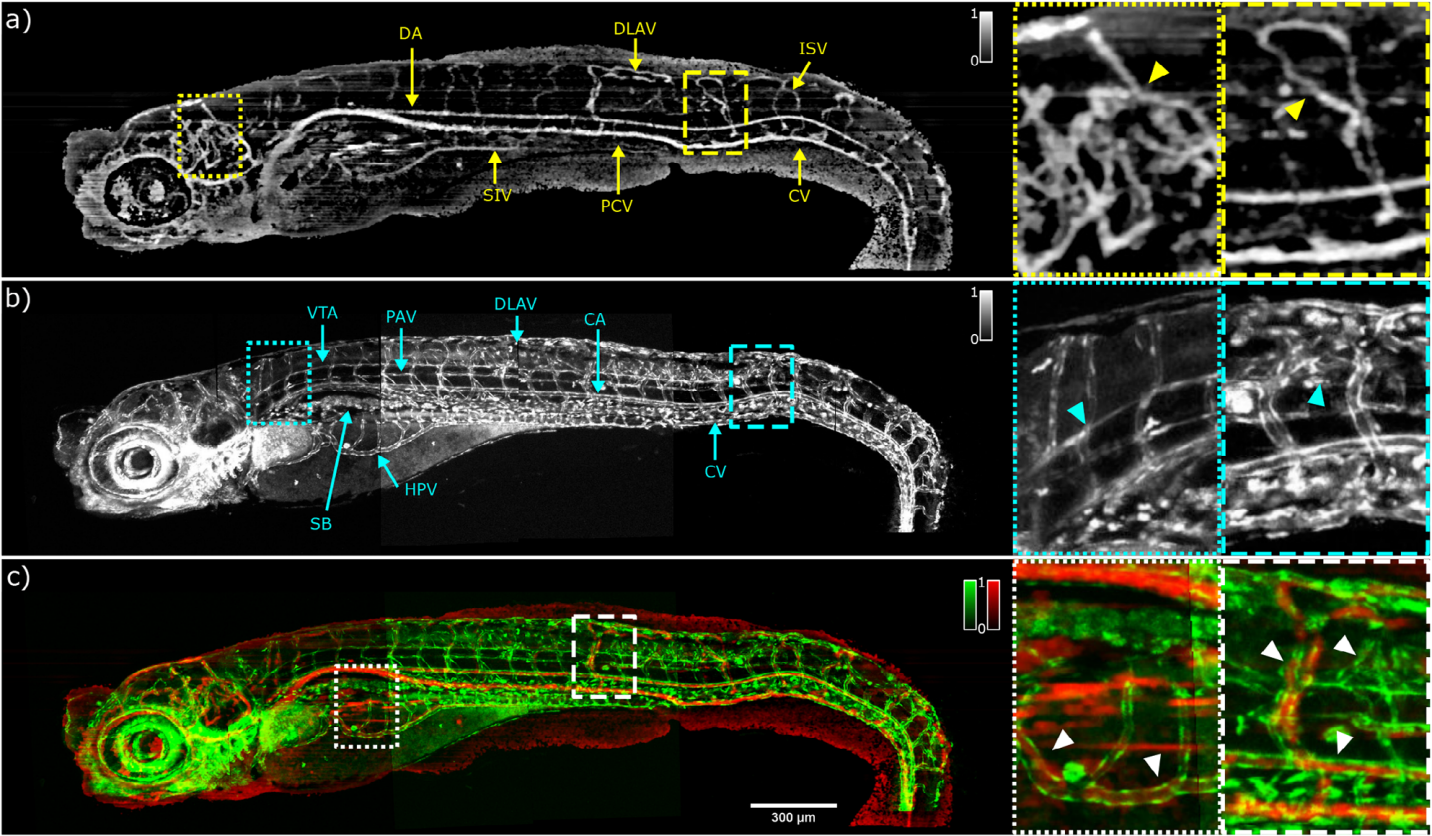
In vivo angiograms of RAS+ zebrafish larvae at 120 hpf. a) en face label‐free OCTA image. The yellow triangles in the yellow dotted rectangle and yellow dashed rectangle highlight the misregulated flows. b) en face confocal GFP fluorescence image. The cyan triangles in the cyan dotted rectangle and cyan dashed rectangle show the uncontrolled growth of blood vessels. c) merged image of the images in panel a) and b): red OCTA; green GFP fluorescence. The white triangles in the dotted and dashed rectangles show the correlation between OCTA and cLSM information. DA, dorsal aorta; SIV, subintestinal vein; ISV, intersegmental vessels; PCV, posterior cardinal vein; DLAV, dorsal longitudinal anastomotic vessel; CA, caudal artery; CV, caudal vein; HPV, hepatic portal vein; SB, swim bladder; VTA, vertebral artery; PAV, parachordal vessel.

To observe the similarities and differences between the OCTA and cLSM data and to verify the origin of the OCTA signals, OCTA and cLSM images are merged in one color‐coded image. The result is shown in Figure 7c. There is a perfect overlap between the two imaging modalities in the en face plane representation. However, an apparent difference in the signals can be observed in the region of the heart and eye region where the cLSM image reveals the strong fluorescence signal. Moreover, the white dotted and dashed ROIs clearly emphasizes the complementary information from the OCTA and cLSM: the GFP signal from the endothelial cells highlights the walls of the vessels but does not reveal any information about the blood flow while the OCTA signal obtains the information of the blood flow of the vessels but does not reveal the full vessel architecture. In contrast to the well‐organized vascular network of the RAS‐zebrafish larvae, the blood vessel network of a RAS+ zebrafish larvae is altered both in the head and trunk of the zebrafish larvae (**Figure** **8**a). Already the bright field image in Figure 4 shows the clear change in morphology in the cancerous phenotype compared to the RAS‐ control. A strong bending of the tail can be observed. The DA, CA, PCV and CV are deformed and bended toward the tail region. The well‐organized ISVs network is not present anymore, instead a tortuous network of blood vessels which lacks the normal hierarchical order is visible. Instead of being all continuous, some parts of the DLAVs and the ISVs form blood flow loops. In addition, the regular spacing between the front and back ISVs is not preserved as shown in the yellow dashed ROI in Figure 8a. However, the vessel network in the trunk region close to the head is comparable to the RAS‐ case. Also, the swim bladder and the subintestinal vein (SIV) are not obviously altered by the cancerous phenotype. The head region shows a chaotic organization of vessels compared to the RAS‐ zebrafish. The DLV is barely visible and its length and thickness is altered. The mesh of blood vessels experiences an uncontrolled growth with an increased number of edges and vertices as shown in the yellow dotted ROI of Figure 8a. Also, the corresponding cLSM image shows distinct changes compared to the RAS‐ control (Figure 8b). The vessel network shows huge variations and differences depending on the ROI and in respect to the RAS‐ case. In the head and trunk regions, where the hyperproliferating cells are developing, a chaotic and irregular number of endothelial cells creates high number of vessels with multiple unusual ramifications as highlighted in the inset of Figure 8b. The length and shape of the ISVs are different compared to the RAS‐ case shown in the second inset. In contrast, in the heart and digestive track regions the well‐organized network is preserved and the intestinal vein and artery can be identified. As for the RAS‐ case, by merging the information carried by the two imaging modalities in a single color‐coded red green image as shown in Figure 8c the complementarity of OCTA and cLSM is revealed. Head and trunk regions show weak correlation between OCTA and fluorescence: OCTA shows much less contrast than the fluorescence image due to a limited blood flow in the cancerous phenotype. The OCTA signal intensity differences are linked to the different decorrelation time between subsequent B‐scans and thus to different flows speed: high flows are linked to high OCTA signal intensity and low flows are linked to low OCTA signal intensity. Most of the image contrast arises from the GFP fluorescence of hyperproliferating endothelial cells not linked to locations with presence of specific vessels as shown in the first inset in Figure 8c. In contrast to the head and trunk regions the vessels in the intestinal tract are well preserved. This behavior is highlighted in the second inset of Figure 8c. The substantial background signals in region where no blood flow is present in the OCTA angiography maps from both RAS‐ and RAS+ zebrafish larvae in Figures 7a and 8a are related to the bulk motion of the living zebrafish larvae.

**Figure 9:**
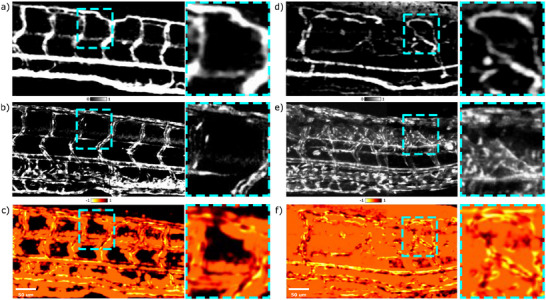
Structural similarity index measure (SSIM) for 120 hpf zebrafish larvae. In vivo OCTA (a) and GFP images (b) of RAS‐ zebrafish larvae and corresponding SSIM results (c). In vivo OCTA (d) and GFP images (e) of RAS+ zebrafish larvae and corresponding SSIM results (f). The SSIM score for the RAS‐ and RAS+ zebrafish are 0.45 and 0.04, respectively. The dashed boxed areas highlight regular ISVs pattern for the RAS‐ case with local SSIM score of 0.33, and vessels looping for the RAS+ case with local SSIM score of 0.02. FOV is 607x394 µm^2^.

Moreover, a representative OCTA of treated larvae with lonafarnib at 1µM and 3µM and with trametinib at 100 nM can be found in Figure S2 (Supporting Information). For more comprehensive visualization of the 3D nature of the OCTA angiograms, we show depth‐resolved color‐coded angiograms in Figure S1 and in Movie 2 (Supporting Information), 3D angiograms for RAS+ and RAS‐ larvae at 120 hpf. Moreover, representative OCTA data of treated larvae with lonafarnib at 1 µM and 3 µM and with trametinib at 100 nM can be found in Figure S2 (Supporting Information). For better comparison and evaluation of the image quality and structural similarities between OCTA and GFP images the structural similarity index (SSIM) was calculated.^[^ ^43^ ^]^ SSIM is particularly valuable because it mimics how humans perceive structural changes in images rather than focusing solely on pixel‐by‐pixel differences like traditional metrics (e.g., mean squared error). For this calculation we have considered a region of interest encompassing six intersegmental vessels. **Figure** **9**a,d shows the OCTA signals for RAS‐ and RAS+ zebrafish, respectively while Figure 9c,e shows the corresponding fluorescence signals for RAS‐ and RAS+ zebrafish, respectively. SSIM maps for RAS‐ and RAS+ zebrafish are shown in Figure 9c,f, respectively. A small local SSIM value appears as white pixels (high level of dissimilarity) and a large value appears as black pixels (high level of similarities). Therefore, regions with small SSIM value correspond to areas where OCTA differs from GFP images. Regions with large SSIM value correspond to regions with high degree of similarity. Indeed, Figure 9c, shows a regular pattern of regions with high degree (black areas) and low degree of similarity (orange and yellow areas), resembling the well ordinated vascular network highlighted by OCTA and GFP fluorescence images of RAS‐ zebrafish. The SSIM map of RAS+ zebrafish (Figure 9f) does not show any pattern of areas with high and low local SSIM values and overall only reveals an increased level of dissimilarity. The SSIM score for the images in Figure 9c,f is 0.45 for RAS‐ zebrafish and 0.04 for RAS+ zebrafish which indicates relative high degree of structural similarity for RAS‐ zebrafish and almost no similarity for RAS+ zebrafish. The boxed dashed areas in Figure 9 highlight regions where the SSIM score indicates high degree of similiarity for the RAS‐ case and no similarities for the RAS+ case. Those areas corresponds to the well ordinated ISVs structure (RAS‐) and a loop formed at the ISV level (RAS+). The local SSIM score for those areas are 0.33 for RAS‐ and 0.02 for RAS+.

### 2.5 Topological Vessel Analysis

**Figure 10:**
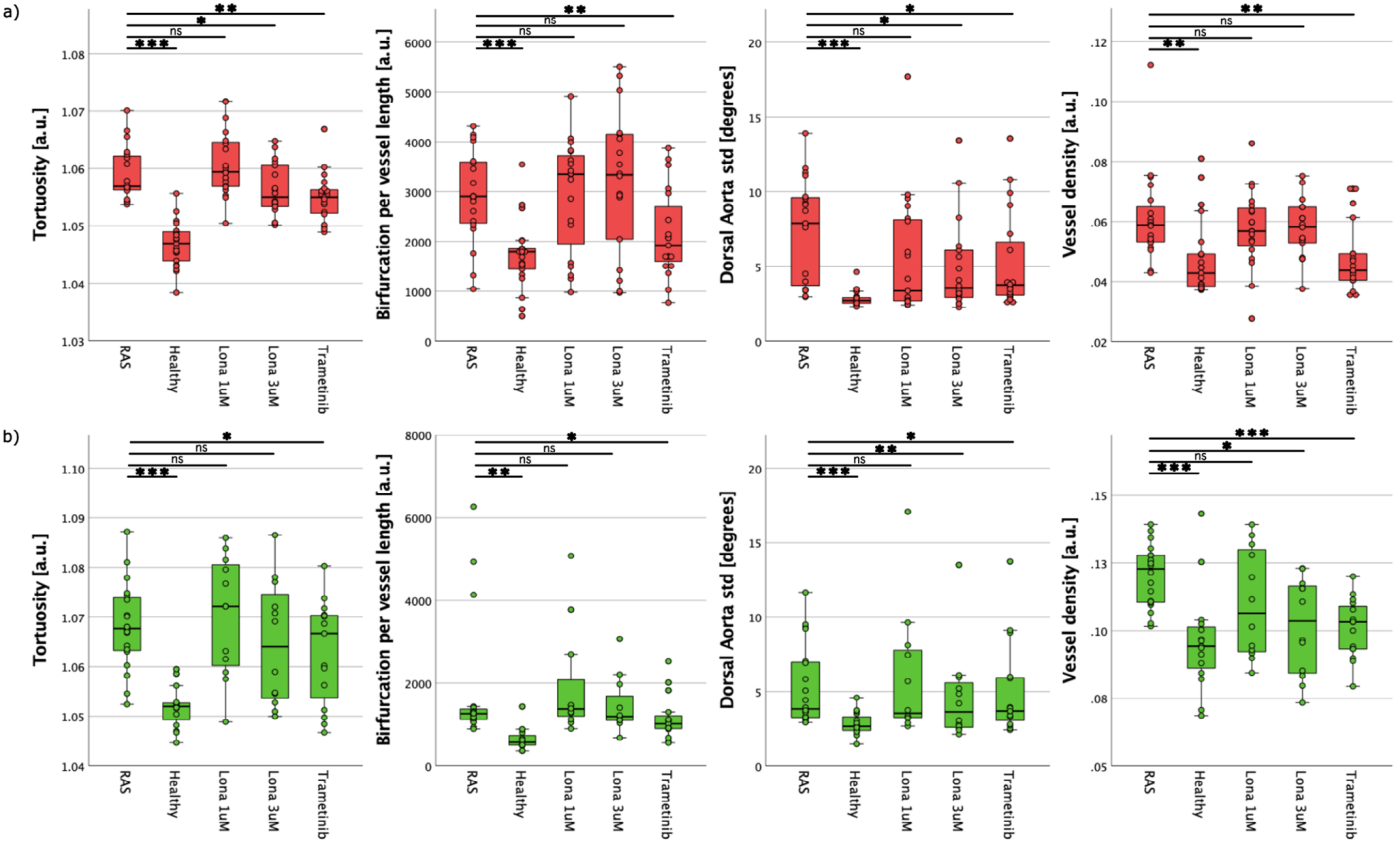
Four most relevant topological metrics describing the vascular and flow network differences between RAS‐, RAS+ larvae. The results of treated larvae with lonafarnib at 1 µM and 3 µM concentrations and with trametinib at 100 nM are shown as well. Significant differences between RAS‐ and RAS+ groups are shown (ns non‐significant, *p< 0.05, **p < 0.01, and ***p < 0.001). The group size per category is n = 20. a) OCTA and b) GFP topological analysis results.

Next we aimed at establishing parameters, which best describe the blood flow and can serve as metrics to score the effects of small compounds toward restoring a normal physiological vascular network. Five parameters from binarized OCTA and GFP en face images were calculated to obtain an objective analysis of the vascular flow network for RAS‐ and RAS+ zebrafish larvae and to reveal distinct differences between the control group, the cancer model and the zebrafish larvae treated with different concentrations of different drugs. The five identified parameters are tortuosity, DA and CA angle standard deviations, vessel density, mean vessel diameter, and bifurcations per vessel length. We used these parameters as biomarkers to differentiate between RAS‐ and RAS+ larvae. The results of the analysis are given in **Table** **1**. The corresponding box plots are shown in **Figure** **10** for the most OCTA significant features describing the differences in the flow network between RAS‐ and RAS+ larvae. The group size per category was fixed to n equal 20 for all five groups.

**Table 1:** Topological analysis of the vascular network in the zebrafish trunk of RAS‐ and RAS+ larvae. Shown are the mean values with the standard deviations in parentheses.

	OCTA			GFP		
	RAS‐	RAS+	*p*‐value	RAS‐	RAS+	*p*‐value
Dorsal Aorta and Caudal Artery angle std [degrees]	2.838 (0.516)	7.475 (3.443)	<0.001	2.854 (0.775)	7.498 (2.708)	<0.001
Tortuosity [a.u.]	1.047 (0.004)	1.059 (0.005)	<0.001	1.052 (0.004)	1.068 (0.008)	<0.001
Bifurcations per vessel length [a.u.]	1724.371 (686.049)	2927.517 (939.673)	<0.001	649.953 (270.403)	1786.601 (1483.802)	0.002
Vessel density [a.u.]	0.048 (0.129)	0.062 (0.015)	0.002	0.096 (0.189)	0.120 (0.113)	<0.001
Mean vessel diameter [µm]	9.187 (1.477)	9.253 (1.677)	0.448	6.329 (0.937)	6.042 (1.883)	0.279

For OCTA, mean vessel diameter values are almost identical between RAS+ and RAS‐ zebrafish larvae (9.253 vs 9.187; p = 0.448), indicating no discriminative power of this parameter between groups. However, the flow pattern in the RAS+ larvae is characterized by a higher tortuosity (1.059 vs 1.047; p < 0.001) and a higher number of bifurcations per vessel length (2927.5 vs 1724.4; p < 0.001). Furthermore, the standard deviation of the alignment angle of the dorsal aorta and caudal artery is significantly higher in RAS+ larvae (7.475 vs 2.838; p < 0.001), and the vessel density is also increased (0.062 vs 0.048; p = 0.002). Overall, four out of the five analyzed OCTA parameters show significant differences, underlining the potential discriminative power (see Table 1 for detailed results). For GFP, mean vessel diameter values show the same trend as OCTA and are almost identical between RAS+ and RAS‐ zebrafish larvae (6.042 vs. 6.329; p = 0.279). In contrast, the vascular pattern in the RAS+ larvae exhibits a higher tortuosity (1.068 vs 1.052) and a larger standard deviation of the alignment angle of the dorsal aorta (7.498 vs 2.854) compared to the RAS‐ larvae, with both differences being highly significant (p < 0.001). Bifurcations per vessel length is also higher in RAS+ larvae (1786.6 vs 650.0; p = 0.002), as is vessel density (0.120 vs 0.096; p < 0.001). In total, four out of the five GFP‐derived parameters show significant differences.

### 2.6 Drug and Dose Efficacy Measurements

The multimodal measurements revealed distinct differences between RAS‐ and RAS+ zebrafish larvae and complementary contrast arising from OCTA and GFP fluorescence imaging. To demonstrate the full potential of our approach as tool for drug screening and detection of subtle and dose dependent changes in the vascular network, we studied the effect of two compounds, i.e. lonafarnib and trametinib, which both act on RAS‐expressing tumor cells. We assumed that blocking RAS‐mediated effects, we would also ameliorate the aberrant angiogenesis. We used the parameters identified in the previous section to evaluate the effects of those small compounds. Thereby, we treated RAS+ zebrafish larvae with 1 and 3 µM lonafarnib and 100 nM trametinib as described in detail in Section 4. We performed OCTA and fluorescence imaging of the three groups of treated zebrafish larvae (120 hpf) followed by the topological analysis from binarized OCTA and GFP en face images such as for RAS+ and RAS‐.

Results are shown in the box plots of Figure 10 for the four most significant OCTA parameters identified in Section 2.5.

In the upper part of Figure 10 OCTA analysis results are shown. Lonafarnib 1 µM treated RAS+ zebrafish larvae reveal information about tortuosity, bifurcation per vessel length, DA angle standard deviation and vessel density mean values similar to the RAS+ zebrafish larvae. At higher doses, Lonafarnib shows higher efficacy and tortuosity, DA angle standard deviation shows statistical significance. Additionally, the effectiveness of trametinib 100 nM is similar to lonafarnib at 3 µM for tortuosity and DA angle standard deviation. Nevertheless, the number of bifurcation per vessel length and vessel density mean value are considerably reduced compared to the zebrafish treated with two different concentrations of lonafarnib reaching a similar value as for the RAS‐ larvae.

In the lower part of Figure 10, GFP analysis results are shown. Lonafarnib 1 µM treated RAS+ zebrafish larvae reveal no significant different mean value for all four parameters than RAS+. At higher doses, lonafarnib shows higher efficacy and all the four parameters have reduced mean values approaching the mean values as for RAS‐ larvae. The effectiveness of trametinib 100 nM is similar to lonafarnib at 3 µM for tortuosity, DA angle standard deviation and vessel density.

## 3 Discussion and Conclusion

We have developed a label‐free non‐invasive optical imaging platform and analysis pipeline that enables detailed insight into vasculature and perfusion of vessels. We applied our system to study angiogenesis in healthy zebrafish and in a cancer model of tumor vascularization. OCTA maps of an in vivo zebrafish cancer model have been generated and correlated to cLSM images. OCTA enables novel and advanced measurements of blood flow dynamics and vascular structure in biological systems, offering label‐free, non‐invasive imaging with high spatiotemporal resolution. In our study, OCTA has been optimized and applied to analyze vascular perfusion in zebrafish models, both healthy (RAS‐) and a cancer model driven by RAS oncogene overexpression (RAS+). The optimization of the inter B‐scan rate was crucial due to the varying red blood cell velocities in zebrafish, particularly as flow dynamics change between healthy and diseased conditions. In healthy zebrafish, the red blood cell velocity is approximately 230 µm/s in the caudal artery and 580 µm/s in the dorsal artery at 120 hours post‐fertilization.^[^ ^44^ ^]^ However, in cancerous zebrafish larvae, red blood cell velocities can drop to just a few µm/s, indicating significant flow impairments. To capture fast and not to miss slow blood flow, the optimal inter B‐scan rate was determined to be 160 ms. According to Richter et al.,^[^ ^45^ ^]^ this rate allows for the visualization of fast‐flowing vessels with high contrast while maintaining sufficient signal‐to‐noise ratio (SNR) to detect slow‐flowing vessels, enabling comprehensive blood flow analysis in complex biological systems, thus making OCTA a powerful tool to investigate the changes in blood flow associated with disease progression and treatment response.

In healthy zebrafish, OCTA revealed consistent blood flow across the vasculature, with a close match between OCTA‐derived blood flow maps and GFP fluorescence imaging of vessel walls. This correlation was highly regular in healthy zebrafish, where both vessel structure and blood flow were well‐matched, as shown by our topological analysis and by a SSIM score of 0.45 indicating an increased level of similarities compared to RAS+. However, in the RAS+ zebrafish cancer model, the vascular network becomes more complex due to aberrant angiogenesis generating new and partially non‐perfused vessels, resulting in an SSIM score of 0.04 between the OCTA and GFP images. This score indicates no similarity between blood flow contrast and endothelial cell contrast. The newly formed vessels are not perfused, leading to a lower SSIM score. If these vessels were perfused, the SSIM score would be higher. The disorganized vascular growth in RAS+ larvae, characterized by new vessels that were either partially or not at all perfused, showcased GFP and OCTA's capability to differentiate between structural vessels and functional blood flow. These differences indicated that a lot of the newly formed vessels are not functional and some of the already established ones loose functionality. This behaviour is described by the vessel density and the bifurcation per vessel length markers in our quantitative topological analysis, where the GFP results show, in both cases, much higher values in the RAS+ zebrafish compared to the OCTA values (**Table** **1**). The high vessel density around the tumor, combined with increased tortuosity and bended DA and CA, was highlighted by OCTA and GFP and used as quantitative markers of pathological angiogenesis. Importantly, the combination of GFP and OCTA analysis helped delineating the differences between the structural formation of vessels and the actual flow of blood within them, a critical aspect of understanding tumor‐related vascular dysfunction. In particular, OCTA's utility extends beyond imaging and has been successfully employed to evaluate the effects of small‐molecule drugs on vascular structure and blood flow. We dissected the effects of two drugs, trametinib and lonafarnib, in the RAS+ zebrafish cancer model. Trametinib, a MEK inhibitor, acts on the downstream pathway of the HRAS oncogene, while lonafarnib inhibits HRAS post‐translational modification, both of which are critical in mediating cancer cell proliferation. OCTA in combination with GFP allowed us to quantitatively assess the impact of these drugs on the aberrant angiogenesis observed in the RAS+ zebrafish. Specifically, we found that treatment with these drugs significantly slowed or even halted the formation of new, dysfunctional vessels. By measuring parameters such as vessel density, CA and DA bending, and tortuosity, we were able to demonstrate that both trametinib and lonafarnib effectively reduced pathological angiogenesis in a dose‐dependent manner. The ability of OCTA to capture real‐time changes in blood flow and GFP to highlight vessel morphology offer a powerful platform for assessing drug efficacy in cancer models, providing an objective, quantifiable measure of treatment outcomes.

The traditional method for assessing blood flow in zebrafish models often involves using transgenic lines that express fluorescent markers in red blood cells (RBCs), such as the *Tg(gata1:dsRed)* ^*sd*2^ line.^[^ ^46^ ^]^ This allows researchers to track individual RBCs moving through the vasculature. However, OCTA offers several advantages over this approach. First, it provides a label‐free method for visualizing blood flow, which eliminates the need for genetic manipulation or exogenous labeling. Second, while traditional methods focus primarily on the movement of red blood cells, OCTA can offer a broader picture by capturing the entire blood flow profile, including areas where flow is impaired or absent. OCTA's ability to reveal regions of impaired perfusion in tumor vasculature highlights its potential as a more comprehensive and less invasive approach. In particular, OCTA can detect regions where vessels are present but not perfused. This makes OCTA particularly useful in studying complex vascular systems, such as those seen in cancer models, where not all vessels are functional.

Despite the promising results obtained with the proposed methodological approach, there are a few limitations to consider. One limitation of our current analysis is that it was based on a maximum intensity projections of the volumetric data, which may result in an overestimation of vessel bifurcations and a loss of depth information. While this approach was chosen for its simplicity and ease of overlap with fluorescence images, future studies could benefit from a full 3D analysis of the vasculature to provide a more accurate representation of vessel networks. Additionally, our sample size was relatively small (20 larvae per group), but the differences observed between the RAS+ and RAS‐ models were visually and qualitatively apparent, suggesting that these results are robust and would likely hold true with larger sample sizes. Another area of potential improvement is the integration of more advanced algorithms for analyzing flow dynamics. For example, the use of variable time‐interval analysis, as seen in retinal vasculature studies,^[^ ^9^ ^]^ could enhance OCTA's ability to capture a wider range of flow speeds, from fast arterial flow to slower venous or capillary flow. This could be particularly useful for studying complex biological systems where different vessels exhibit varying flow rates, such as during development or in disease states like cancer.

In conclusion, the proposed approach provides a novel and powerful platform for measuring blood flow and vascular structure in biological systems, offering significant advantages over traditional imaging methods. By combining OCTA with fluorescence imaging, we can obtain a comprehensive view of both vessel structure and function, allowing for a more detailed understanding of normal and pathological vascular dynamics. In our study, OCTA and GFP imaging revealed critical differences between healthy zebrafish and a cancer model, highlighting its ability to detect impaired perfusion and dysfunctional angiogenesis. Moreover, the proposed approach in evaluating drug efficacy underscores its potential as a valuable tool in preclinical drug screening and personalized medicine. With further optimization and integration of advanced analysis techniques, it could become a key method for studying vascular networks in a wide range of biological and disease models.

## 4 Experimental Section

### Zebrafish Model and Maintenance

In this study, angiogenesis is investigated in a transgenic zebrafish (*Danio rerio*) cancer model. Zebrafish were maintained according to standard protocols^[^ ^47^, ^48^ ^]^ and the guidelines of the local authorities under licenses GZ:565304/2014/6 and GZ:534619/2014/4. Transgenic *Tg(gata1:dsRed)* ^*sd2*^ zebrafish larvae were used to visualize erythrocytes. Transgenic zebrafish strains *Tg(fli1a:EGFP)^*y*1^/Et(SP8b:KalTA4‐1xUAS:mCherry)/Tg(H2BCFP:UAS: HRAS^*G*12*V*^)^*vi*014^* were used to demarcate endothelial cells with green fluorescent protein while targeting oncogene (HRAS^*G*12*V*^) expression to the CNS.^[^ ^41^, ^49^ ^]^ This leads to hyperproliferation of neural cells within the brain and the spinal cord in zebrafish larvae, inducing head and tail deformation. Zebrafish larvae, from RAS+ and RAS‐ zebrafish, were manually dechorionated and maintained in E3 medium at 28°C under standard conditions. Pigmentation was inhibited by adding 1‐phenyl 2‐thiourea (PTU, CAS Number:103‐85‐5, Sigma Aldrich) to the E3 fish medium at 24 hpf. At 120 hpf, zebrafish still vital but showing strong RAS phenotype were picked and transferred into a small petridish. At this point the selected larvae were prepared for the in vivo imaging session as follows: first, larvae were lightly anesthetized in E3 fish medium with Tris‐HCl buffered 130 mg/L tricaine (CAS Number 886‐86‐2, Sigma‐Aldrich); second, each larva was immersed in 0.7% Phytagel at 32°C (CAS Number 71010‐52‐1, Sigma Aldrich) to prevent movement, and third, placed in a sterilized coverslip glass bottom dish and manually positioned sidewards perpendicular to the optical axis. Humidity level of the Phytagel was preserved by adding E3 medium to the glass bottom dish after gelification. The use of 0.7% low melting Phytagel ensured reduced optical scattering of the laser light in comparison to the classical ultra‐low gelling agarose and thus an increased image contrast.

### Drug Screening

The effects of trametinib (CAS Number 871700‐17‐3, MedChemExpress) and lonafarnib (CAS Number 193275‐84‐2, MedChemExpress) were investigated which were prepared as 10mM DMSO stocks and added when larvae were 26 hpf old to E3 fish medium to reach indicated concentrations.

### OCT imaging

In vivo volumetric OCTA data sets of the zebrafish larvae were acquired using a custom‐built spectral domain OCT (SD‐OCT) system implemented in an upright microscope (Nikon Eclipse E400). The detailed description of the system can be found in Ref. [50]. In brief, the SD‐OCT imaging system was performed by means of a home‐build broadband Ti:sapphire laser with a full width half maximum bandwidth of 150 nm centered at ∼800 nm. A 12‐bit CCD‐based line scan camera with a maximum line rate of 70 kHz and 2048 pixels (AViiVA Atmel EM4CL 2014, Essex, UK) was interfaced to the spectrometer, providing an imaging depth of 1.3 mm in air. The laser beam was focused onto the sample through a 4x Nikon microscope objective with 0.1 numerical aperture (NA) and a working distance of 30 mm. With such configuration, the SD‐OCT system provided a measured lateral resolution of ∼4 µm and an axial resolution of ∼2.9 µm in air. An OCT sensitivity of 98 was achieved dB with 500 µW incident power on the zebrafish. In vivo data acquisition was performed over a rectangular window of 3000 x 600 pixel corresponding to a FOV of 4.2 x 0.8 mm. Angiography based on the phase contrast was obtained from the acquired OCT volumes.^[^ ^51^ ^]^ A motorized translation stage (Physik Instrumente – C‐867) facilitated the navigation. After re‐slicing of the volume a stack of C‐images (xy‐plane) was generated and depth resolved information was obtained via color coding. For OCTA data acquisition, the phase variance optical coherence technique was applied and multiple B‐scans were acquired at each position of the slow axis (modulated B‐scans – BM‐scans). The phase difference was calculated between consecutive B‐scans taken at the same transversal location from the acquired OCT volumes.^[^ ^51^ ^]^ The total phase change contained contributions from the phase change from the moving blood cells and other moving scatterers, phase noise due to bulk motion and phase noise from the light source and system as well as other parameters contribution to phase errors. Since the acquisition time is depending on the flow speed and prolonged for OCTA data sets, the system was optimized in terms of scanning density, image quality, acquisition time and resolvable flow speeds.

In anesthetized larvae the average peak and minimum aortic flow velocities were reported to be ∼850 and 210 µm/s in 120 hpf.^[^ ^37^ ^]^ However, the reported blood flow varies depending on the system parameters such as speed and sensitivity of the measurement device and anesthesia, embedment method or cooling of the organisms. Velocities are also strongly connected with age. Measurements of oxygen concentrations have shown that conditions go hypoxic after 5 min in larvae 120 hpf embedded in agarose gel.^[^ ^44^ ^]^ For these reasons, the zebrafish larvae were kept less than 5 min embedded. Since each A‐scan consisted of 1024 points and 3000 A‐scans contributed to a B‐scan, up to 10 repetitions were recorded at each position before shifting to the next slow‐axis location with a total number of 600 B‐scans over a region of 0.8 mm. A custom‐based Matlab program acquired and saved the data while another Matlab program was used for post‐processing and phase difference calculations. Phase unwrapping was applied on the extracted phase difference values, then the bulk noise was removed and the intensity data were used to threshold and mask noise pixels. The noise was further reduced by applying a median filter and the en face angiograms with a size of 4.2x0.8 mm were created with a maximum projection of the angiography data in axial direction.

### Confocal Fluorescence Imaging

GFP fluorescence imaging was performed as complementary method and gold standard to examine and describe the vascular anatomy of the zebrafish and to benchmark our OCTA images. The genetically modified zebrafish model used in this work, expresses GFP in endothelial cells thus highlighting the interior surface of blood vessels and lymphatic vessels. Hence, the GFP contrast in this model is linked to blood vessels and was used to compare the contrast from cLSM with OCTA contrast in living zebrafish larvae. Moreover, dsRed fluorescence imaging was performed labeling erythrocytes to correlate the OCTA signal to the signal of the movement of erythrocytes. Images in Figure 1 were recorded on a Leica confocal microscope SP5 using a 25x water objective. Stand stills from the movie shown in Figure 2 were recorded with a 40x water objective. Larva GFP and dsRed imaging for comparative analysis with OCTA signal was performed by using a Leica confocal microscope SP8. For comparison reasons we have used a HC PL FLUOTAR 10x/0.30 dry objective which gives a rather similar optical performances compared to the OCTA images. A series of 39 depth resolved images, corresponding to a total depth of 304 µm was recorded and used to generate en face images of the zebrafish vasculature. The resulting images had a pixel size of 1.14 µm in the en face plane and 8 µm in the depth direction.

### Data Processing and Presentation

To construct a label‐free map of blood flow, OCTA images were calculated from OCT complex data by quantifying the phase correlation between consecutive OCT cross‐sectional scans (B‐scans) taken at the same cross‐sectional location. Hence, static regions or regions with negligible motion did not produce a phase change in the consecutive OCT B‐scans taken at the same location. On the other side, all moving particles created a phase difference between consecutive B‐scans. This phase value difference is directly linked to pixel intensity. To reduce the noise in the OCTA images numerical filtration was performed. The data filtering pipeline includes a 3D directional filter plus a 2D median filter (on the en‐face plane) with 3 pixels radius to enhance the vessels contrast followed by a manual thresholding. At this point, en face OCTA and GFP projections by maximum intensity projection were created. These projections intrinsically encode the 3D information used for the topological analysis. In order to visualize similarities and differences between the vessel network highlighted by OCTA and GFP a merged image was produced as shown in Figures 7c and 8c.

### Structural similarities

Vascular similarity and dissimilarities between OCTA and GFP maps were calculated in an objective way using the structural similarity index measure (SSIM). SSIM is a metric used to assess the similarity between two images, based on their structural information where structure refers to the spatial arrangement of pixel intensities in the OCTA and GFP images with contrast arising from blood flow and endothelial cells. Unlike traditional methods, such as mean squared error or peak signal‐to‐noise ratio, which primarily focus on pixel differences, SSIM considers how human vision perceives structural changes. Thus, the goal of SSIM is to quantify image quality by comparing three main image components: luminance, contrast, and structure. Each of these components is calculated separately, and then combined to give an overall SSIM score. The SSIM index produces a value between –1 and 1, where 1 indicates identical structural similarity between the images, 0 indicates no similarity and –1 indicates perfect anti‐correlation. The builtin SSIM function was used in Matlab for this calculation and the results presented in form of images with an inverted hot colormap where black color corresponds to SSIM score of 1 and white color corresponds to SSIM score of –1. Levels of no similarities (score of 0) are indicated with orange color.

### Vascular Network Analysis

While the ability to visually track associations between OCTA images and pathology is important to evaluate the importance of OCTA, it is equally important to have the ability to track such correlations using quantifiable measures for objective comparison. Image data analysis was performed on the specific ROIs of OCTA en face images to highlight these associations following the workflow shown in Figure 3. The selected ROI cover a region of 6 ISVs centered on the gut location and including DA, CV and DLAV. Vessels in the ROI were manually segmented. The binary angiograms were skeletonized and analyzed with the help of scikit‐image^[^ ^52^ ^]^ and skan^[^ ^53^ ^]^ libraries. Analysis of the vascular network in OCT angiograms was performed by calculating five metrics which could be related to the RAS+ phenotype: the first parameter is the vessel tortuosity and is defined as the ratio between the distance along the vessels and the Euclidean distance between the two endpoints of the examined segment.^[^ ^54^ ^]^ A larger tortuosity value indicates a more curved vessel segment, whereas a value of 1.0 corresponds to a straight vessel segment. Tortuosity was calculated for overlapping vessel segments of a fixed length of 20 pixels and averaged over all segments in the angiogram. The second parameter is related to the distribution of vessel orientations: angular orientation in image space, measured in degrees, was determined for all vessel positions. A Gaussian function was then fit to the histogram of the angle data to model the DA and CV parallel to the spine. The sigma parameter of the fitted Gaussian function is used as the second metric. A larger sigma corresponds to more randomly aligned vessels, whereas a smaller sigma indicates a more regular alignment with many straight and parallel vessels. Vessel density is the third calculated metric and represents the percentage of pixels in the ROI that shows vessels. Sato filtering of curvilinear structure and contrast limited adaptive histogram equalization were applied to the en‐face OCTA images.^[^ ^55^, ^56^ ^]^ Automated thresholding, using Otsu's method, produced a binary image where the percentage of white pixel respect to black pixels represent the vessel density parameter.^[^ ^57^ ^]^ Then, mean vessel diameter was calculated as a ratio between vessel density and the vessel length normalized to the ROI area. The vessel length is calculated from the skeletonized binary image following the principle in Ref. [58]. The fifth parameter is the number of bifurcation normalized in respect to the vessel length. Statistical analysis was performed for RAS+ and RAS‐ zebrafish larvae (n = 20) for all the metrics. Shapiro–Wilk W‐test for normality distribution was initially performed.^[^ ^59^ ^]^ The null hypothesis at significance level of 0.05 was in all cases not rejected. Furthermore, *t*‐tests were performed, thereby p‐values smaller than 0.05 were considered as significantly different. All statistical analyses were performed in SPSS (IBM SPSS Statistics, Version 28).

## Conflict of Interest

The authors declare no conflict of interest.

## Supporting information

Supporting Information

## Data Availability

The data that support the findings of this study are available from the corresponding author upon reasonable request.
