# Supplementary material for: Multimodal Investigation of Angiogenesis and Its Prevention by Small Compounds in a Zebrafish Cancer Model
Source: Adv Sci (Weinh). 2025 Jun 25;12(33):e15176. doi: 10.1002/advs.202415176 (PMC12412526; doi:10.1002/advs.202415176)
Supplement: Supplementary file 1 — Supporting Information [file ADVS-12-e15176-s001.pdf]

ADVANCED SCIENCE
Open Access

Supporting Information

for *Adv. Sci.*, DOI 10.1002/advs.202415176

Multimodal Investigation of Angiogenesis and Its Prevention by Small Compounds in a Zebrafish Cancer Model

Marco Andreana, Ryan Sentosa, Caterina Sturtzel, Martin Pfister, René Werkmeister, Anna Schmitt, David Traver, Rainer Leitgeb, Wolfgang Drexler, Martin Distel and Angelika Unterhuber**

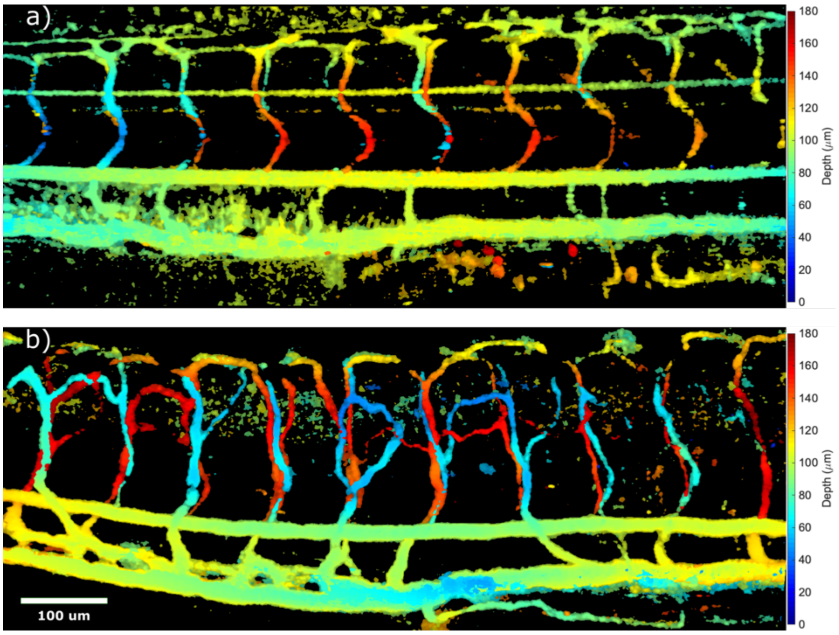

Figure S1: Depth-resolved color-coded angiograms for a) RAS- and b) RAS+ zebrafish larvae at 120 hpf.

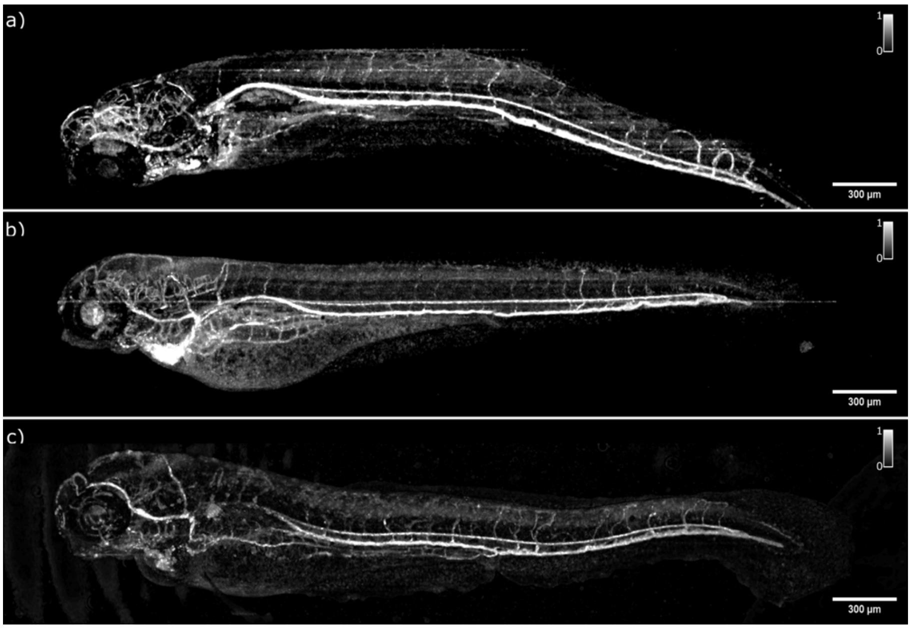

Figure S2: In vivo OCTA angiogram of a) lonafarnib at 1 μM, b) lonafarnib at 3 μM c) tramentinib at 100 nM zebrafish treated larvae at 120 hpf.
